# Magnetization transfer ratio of the sciatic nerve differs between patients in type 1 and type 2 diabetes

**DOI:** 10.1186/s41747-023-00405-1

**Published:** 2024-01-09

**Authors:** Christoph M. Mooshage, Lukas Schimpfle, Dimitrios Tsilingiris, Zoltan Kender, Taraneh Aziz-Safaie, Anja Hohmann, Julia Szendroedi, Peter Nawroth, Volker Sturm, Sabine Heiland, Martin Bendszus, Stefan Kopf, Johann M. E. Jende, Felix T. Kurz

**Affiliations:** 1 Department of Neuroradiology, Heidelberg University Hospital, Im Neuenheimer Feld 400, Heidelberg, 69120 Germany; 2 Department of Endocrinology, Diabetology and Clinical Chemistry (Internal Medicine 1), Heidelberg University Hospital, Heidelberg, Germany; 3 German Center of Diabetes Research, associated partner in the DZD, Munich-Neuherberg, Germany; 4 Institute for Diabetes and Cancer (IDC), Helmholtz Diabetes Center, Helmholtz Center, Munich, Neuherberg, Germany; 5 Department of Neurology, Heidelberg University Hospital, Heidelberg, Germany; 6 Joint Heidelberg-IDC Translational Diabetes Program, Inner Medicine 1, Heidelberg University Hospital, Heidelberg, Germany; 7 Division of Experimental Radiology, Department of Neuroradiology, Heidelberg, Germany; 8 German Cancer Research Center, Heidelberg, Germany

**Keywords:** Diabetes mellitus (type 1 type 2), Diabetic neuropathies, Magnetic resonance imaging, Polyneuropathies, Sciatic nerve

## Abstract

**Background:**

Previous studies on magnetic resonance neurography (MRN) found different patterns of structural nerve damage in type 1 diabetes (T1D) and type 2 diabetes (T2D). Magnetization transfer ratio (MTR) is a quantitative technique to analyze the macromolecular tissue composition. We compared MTR values of the sciatic nerve in patients with T1D, T2D, and healthy controls (HC).

**Methods:**

3-T MRN of the right sciatic nerve at thigh level was performed in 14 HC, 10 patients with T1D (3 with diabetic neuropathy), and 28 patients with T2D (10 with diabetic neuropathy). Results were subsequently correlated with clinical and electrophysiological data.

**Results:**

The sciatic nerve’s MTR was lower in patients with T2D (0.211 ± 0.07, mean ± standard deviation) compared to patients with T1D (T1D 0.285 ± 0.03; *p* = 0.015) and HC (0.269 ± 0.05; *p* = 0.039). In patients with T1D, sciatic MTR correlated positively with tibial nerve conduction velocity (NCV; *r* = 0.71; *p* = 0.021) and negatively with hemoglobin A1c (*r* =  − 0.63; *p* < 0.050). In patients with T2D, we found negative correlations of sciatic nerve’s MTR peroneal NCV (*r* =  − 0.44; *p* = 0.031) which remained significant after partial correlation analysis controlled for age and body mass index (*r* = 0.51; *p* = 0.016).

**Conclusions:**

Lower MTR values of the sciatic nerve in T2D compared to T1D and HC and diametrical correlations of MTR values with NCV in T1D and T2D indicate that there are different macromolecular changes and pathophysiological pathways underlying the development of neuropathic nerve damage in T1D and T2D.

**Trial registration:**

https://classic.clinicaltrials.gov/ct2/show/NCT03022721. 16 January 2017.

**Relevance statement:**

Magnetization transfer ratio imaging may serve as a non-invasive imaging method to monitor the diseases progress and to encode the pathophysiology of nerve damage in patients with type 1 and type 2 diabetes.

**Key points:**

• Magnetization transfer imaging detects distinct macromolecular nerve lesion patterns in diabetes patients.

• Magnetization transfer ratio was lower in type 2 diabetes compared to type 1 diabetes.

• Different pathophysiological mechanisms drive nerve damage in type 1 and 2 diabetes.

**Graphical Abstract:**

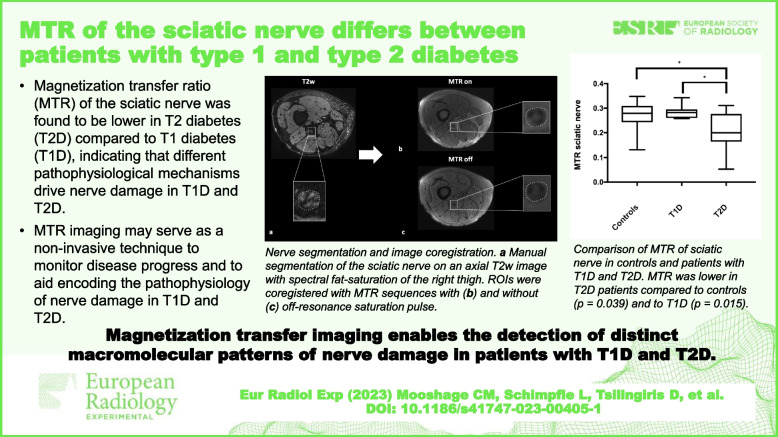

## Background

Distal symmetric neuropathy (DSN) is one of the most frequent complications of diabetes, affecting about half of the patients during the course of the disease [1]. Despite its economical and epidemiological impact, the pathophysiological mechanisms of DSN in diabetes mellitus type 1 (T1D) and diabetes mellitus type 2 (T2D) remain uncertain [2, 3]. However, the current body of literature provides evidence that distinct pathophysiological pathways are responsible for the development of DSN in patients with T1D and T2D [4, 5].

Since experimental animal models of diabetes mellitus fail to replicate all the complex pathophysiological mechanisms of human disease in one model [6], *in vivo* studies using magnetic resonance neurography (MRN) offer a promising technique to gain new insights into the pathophysiology of both entities. So far, diffusion-weighted and T2-weighted as well as dynamic contrast-enhanced sequences have been used to examine DSN [7–13]. In that regard, diffusion weighted imaging in terms of diffusion tensor imaging MRN embodies one of the main sequences in studies of peripheral nerve imaging in general [14, 15], and specifically in patients with T1 and T2D [9, 11, 16]. This approach, among others, enabled the detection of novel biomarkers of peripheral nerve damage in patients with T2D, such as high-sensitivity Troponin T [17].

Meanwhile, the application of T2-weighted MRN sequences demonstrated that the dominating type of fascicular nerve lesion in DSN patients differs between T1D and T2D [7]. While the amount of nerve lesions in T1D was associated with poor glycemic control, nerve lesions of T2D were associated with parameters of lipid metabolism [7], which is in line with the results of large clinical trials in patients with T1D and T2D [4, 5]. Since the exact molecular mechanisms behind the development of DSN in T1D and T2D remain unknown to date, the application of magnetization transfer ratio (MTR) imaging may offer new insights into the pathophysiology of DSN by assessing the nerves macromolecular composition *in vivo*. Due to the short T2 time of protons bound to macromolecules, these protons do not contribute to the signal of conventional MRI sequences [18].

However, MTR imaging enables analysis of the composition of macromolecules in a specific tissue by visualizing the concentration of protons bound to macromolecules like myelin [19]. MTR imaging uses two identical MR sequences, one with and one without an off-resonance saturation pulse, that are applied to selectively saturate protons bound to macromolecules. This can be achieved because the bound protons possess a broader bandwidth of Larmor frequencies compared to free-water protons [20]. After highly selective excitation of the protons bound to macromolecules, spin exchange processes between these protons and the free-water protons result in a decreased longitudinal magnetization, which can be measured and then used to calculate MTR as the quotient of the difference between signal intensities without and with off-resonance saturation and the signal intensity without off-resonance saturation [21].

Previous studies applied MTR imaging in several diseases of the central and peripheral nervous system, the former especially in patients with multiple sclerosis [22]. In patients with multiple sclerosis, a decline of white matter MTR is associated with increased pathomorphological and neurological damage. Meanwhile, in peripheral nerves, MTR imaging was not able to demonstrate an age associated decline of MTR [23] but also that higher levels of neurological deficits are accompanied by lower MTR values thereby demonstrating the value of MTR as a quantitative imaging biomarker in peripheral nerve disorders [24–26]. As MTR allows the quantitative assessment of cerebral and nerval structures, previous studies demonstrated the detection of subclinical pathomorphological alteration in the peripheral and central nervous system [22, 26].

MTR imaging of peripheral nerves has not been performed in T1D and T2D patients yet. The objective of this study was therefore to investigate potential differences of the sciatic nerve’s MTR and correlation with clinical and electrophysiological parameters in order to evaluate the contribution of potential risk factors to nerve damage in in both entities.

## Methods

### Study design and participants

This study was approved by the ethics committee of Heidelberg University Hospital (HEIST-DiC, clinicaltrials.gov identifier NCT03022721, local ethics number S-383/2016), and written informed consent was obtained from all participants. All participants were screened and recruited at the outpatient clinic of the Department of Endocrinology at Heidelberg University Hospital where all clinical, serological, and electrophysiological examinations took place. MRN and image processing was performed in the Department of Neuroradiology at Heidelberg University Hospital. Fifty-two study participants (14 healthy controls (HC), 10 T1D patients, and 28 T2D patients) were enrolled in this prospective single-center study between June 2018 and March 2020. HC were characterized by the absence of any kind of medical condition predisposing for peripheral neuropathy and the absence of any systemic or chronic diseases. Inclusion and exclusion criteria are summarized in Table 1.

**Table 1:** Summary of inclusion and exclusion criteria

Inclusion criteria	Exclusion criteria
Age > 18 years	Pregnancy
Type 1 or type 2 diabetes	Contraindication for MRI
	History of lumbar surgery or disk protrusion
	History of myocardial infarction, coronary heart disease or heart surgery
	Risk factors for neuropathy aside from diabetes Alcoholism Hypovitaminosis Malignant disease Previous or ongoing exposure to neurotoxic agents
	Chronic neurological diseases Parkinson’s disease Multiple sclerosis Restless legs syndrome
	Estimated glomerular filtration rate > 60 mL/min

In line with Gibbon’s criteria [27], diagnosis of DSN was given with a neuropathy disability score (NDS) ≥ 3. Accordingly, four of the T1D patients and ten of the T2D patients were diagnosed with DSN. To preclude differences of sciatic nerve MTR to be caused by typical confounding factors of nerve damage and changes of MTR, groups were matched for hemoglobin A1c (HbA1c), sex, body mass index (BMI), and age [28], so that there were no significant differences between the three groups regarding these parameters.

### Clinical and electrophysiologic examination

A detailed medical history was taken for every participant including an examination of neuropathic symptoms comprising the NDS and the neuropathy severity scale (NSS) as issued by the German Diabetes Association [29]. Blood was drawn in fasting state followed by an immediate analysis by the central laboratory of Heidelberg University Hospital. All electrophysiological studies were conducted on the patients’ right leg by two trained medical technical assistants with more than 6 years of experience in electrophysiological assessments on patients with diabetes maintaining a skin temperature of 32 °C throughout the examination. The electrophysiological examination included the assessment of nerve conduction velocities (NCVs) of tibial, peroneal, and sural nerve, distal motor latencies (DMLs) of the tibial and peroneal nerve, compound muscle action potentials (CMAPs) of the tibial and peroneal nerve, and sensory nerve action potentials of the sural nerve.

### MRI protocol

High-resolution 3-T MRN of the right thigh (Magnetom Tim TRIO, Siemens Healthineers, Erlangen, Germany) was performed at the Department of Neuroradiology at Heidelberg University Hospital using a 15-channel transmit-receive extremity coil. The applied sequences were centered to the sciatic nerve bifurcation at distal thigh level and applied according to the following protocol.

1. One axial high resolution T2-weighted turbo spin echo two-dimensional sequence with spectral fat saturation of the right mid-thigh and the following parameters: repetition time 5,970 ms; echo time 55 ms; field of view 160 × 160 mm^2^; matrix size 512 × 512; slice thickness 4 mm; interslice gap 0.35 mm; voxel size 0.3 × 0.3 × 4.0 mm^3^, number of excitations: 3; images: 24.
2. Two axial proton density-weighted gradient echo sequences, with and without an off-resonance saturation pulse (Gaussian envelop, duration 9,984 μs, frequency offset 1,200 Hz), applied with the exact same field of view and the exact same slice position and the following parameters: repetition time 46 ms; echo time 12.3 ms; flip angle 7°; field of view 200 × 176 mm^2^; matrix size 256 × 256; slice thickness 4 mm, bandwidth 370 Hz/pixel; distance factor 20%; voxel size 1.3 × 1.3 × 4.0 mm^3^; slices: 24, acquisition time 2:17 s:min.

### Image analysis

**Fig. 1:**
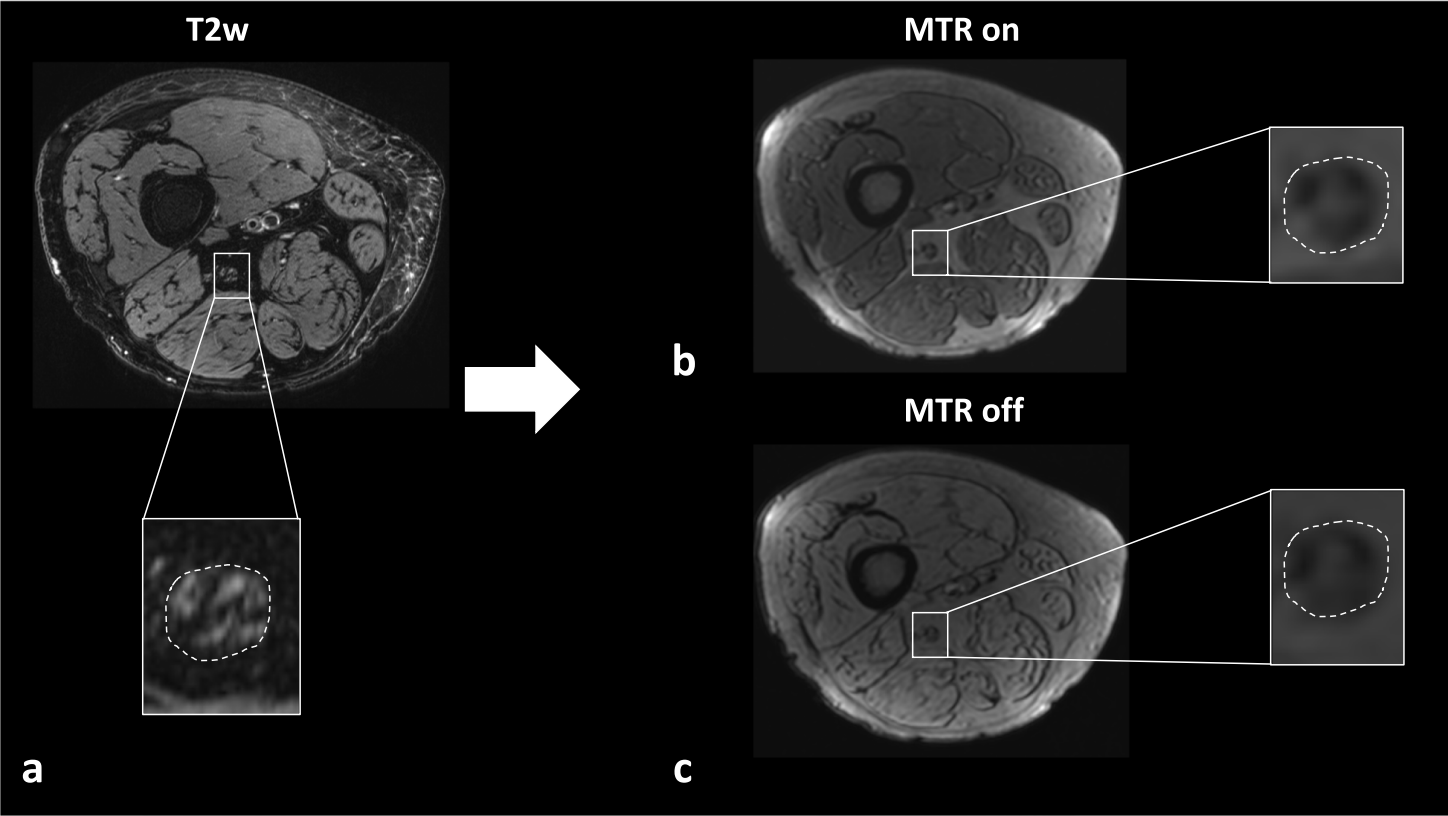
Process of nerve segmentation and image coregistration. **a** Manual segmentation of the sciatic nerve was performed on an axial T2-weighted sequence with spectral fat-saturation (T2w) of the right thigh. Regions of interest were then coregistered with magnetization transfer ratio (MTR) sequences with (**b**) and without (**c**) off-resonance saturation pulse

Image pseudonymization was conducted before analysis and observers were blinded to all clinical data. To ensure precise anatomical segmentation of the sciatic nerve as the region of interest, manual segmentation was performed on the axial T2-weighted sequence by two trained neuroradiologists (C.M.M., F.T.K.) with 5 and 10 years of experience in MRN, respectively, using ImageJ [30]. A custom-written MATLAB code (MathWorks, Natick, MA, USA, R2020b) was applied to conduct semiautomatic coregistration of the created regions of interest to the MTR images with and without off-resonance saturation using affine transformations [31], and to calculate MTR of the sciatic nerve as:

$$\mathrm{MTR}=100\ ({S}_{0}-{S}_{1})/{S}_{0}$$

where *S*_0_ and *S*_1_ represent the signal intensity without and with off-resonance saturation pulse, respectively. MTR was first calculated separately for each image slice whereby only the ten central image slices of each image stack were included into analysis to avoid artifacts or inhomogeneities caused by the *B*_1_ field. Values were then averaged to obtain a mean value for each patient. The process of image coregistration is illustrated in Fig. 1.

### Statistical analysis

MATLAB 7.14.0.0739 (R2012a) and GraphPad Prism 7 were used for all statistical analyses. To test for Gaussian normal distribution, the D’Agostino-Pearson omnibus normality test was applied. If a Gaussian normal distribution was given, *t*-tests were used for comparisons of two groups, one-way ANOVAs were used for comparisons of more than two groups, and Pearson correlation coefficients were used for correlation analysis. If data did not follow Gaussian distribution, the Mann–Whitney *U* test was used for comparisons of two groups, and the Kruskal–Wallis test with post hoc Dunn correction was used for multiple comparisons of more than two groups, and nonparametric Spearman correlation was used for correlation analysis. The level of significance was defined at *p* < 0.05 for all tests.

## Results

### Group comparisons of clinical and demographical data

Of the 52 subjects who took part in this study, 14 were HC (8 women, 6 men), 10 were T1D patients (5 women, 5 men), and 28 T2D patients (14 women, 14 men). Between the groups, there were no significant differences for age, sex, and BMI (*p* ≥ 0.086). In addition, no differences for NDS and NSS scores between HC and patients with T1D and T2D could be found. Disease duration was longer in T1D patients than in T2D patients (29.9 years ± 18.3 [mean ± standard deviation] *versus* 9.7 years ± 9.3, respectively; *p* = 0.002).

### Group comparisons of serological and electrophysiological parameters

HbA1c was higher in T1D patients compared to T2D patients (58.6 mmol/mol ± 9.8 [mean ± standard deviation] *versus* 50.3 mmol/mol ± 8.7; *p* = 0.009), while both T1D and T2D patients had higher HbA1c values than HC (37.0 mmol/mol ± 6.0; *p* < 0.001 for both comparisons). Sural nerve conduction studies were incomplete in three T1D patients and in ten T2D patients due to severe DSN. The remaining measurements were complete. No differences could be found for sural NCV and SNAP, peroneal NCV, CMAP, and DML and for tibial NCV, CMAP, and DML (*p* ≥ 0.105).

### Group comparison of MTR

**Fig. 2:**
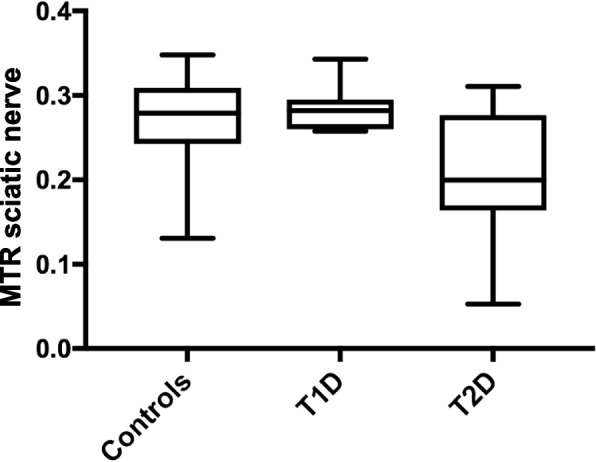
Group comparison of magnetization transfer ratio (MTR) of sciatic nerve in controls (MTR 0.269 ± 0.039, mean ± standard deviation) and patients with type 1 diabetes (T1D, MTR 0.285 ± 0.027) and type 2 diabetes (T2D, MTR 0.211 ± 0.071). MTR was lower in T2D patients compared to controls (*p* = 0.039) and to T1D (*p* = 0.015)

T2D patients (0.211 ± 0.071 [mean ± standard deviation]) had significantly lower MTR values of the sciatic nerve than T1D patients (0.285 ± 0.027; *p* = 0.015) or HC (0.269 ± 0.053; *p* = 0.039). See Fig. 2 for graphic illustration of MTR values and Table 2 for a detailed summary of all group comparisons on demographic, electrophysiological, serological, and MTR data.

**Table 2:** Group comparisons of demographic, serologic, clinical, electrophysiologic, and imaging data of all study participants

	HC	T1D	T2D	*p*-value	*p*-value HC *versus* T1D	*p*-value HC *versus* T2D	*p*-value T1D *versus* T2D
Age (years)	57.8 ± 7.1	53.9 ± 7.2	59.5 ± 8.2	0.151^a^	0.408^c^	0.492^c^	0.152^c^
BMI (kg/m^2^)	26.5 ± 3.5	26.9 ± 5.2	29.2 ± 3.7	0.086^a^	0.828^c^	0.135^c^	0.233^c^
Diabetes duration (years)	NA	29.9 ± 18.3	9.7 ± 9.3	0.002^e^	NA	NA	NA
Sex (w/m)	8 w/6 m	5 w/5 m	14 w/14 m	0.903^d^	> 0.999^b^	> 0.999^b^	> 0.999^b^
MTR	0.269 ± 0.053	0.285 ± 0.027	0.211 ± 0.071	0.004^d^	> 0.999^b^	0.039^b^	0.015^b^
HbA1c (mmol/mol)	37.0 ± 6.0	58.6 ± 9.8	50.3 ± 8.7	< 0.001^a^	< 0.001^c^	< 0.001^c^	0.009^c^
eGFR (mL/min)	92.3 ± 15.4	90.9 ± 20.1	87.2 ± 15.3	0.697^d^	> 0.999^b^	> 0.999^b^	> 0.999^b^
NDS	1.4 ± 1.3	2.9 ± 2.9	2.8 ± 2.7	0.175^a^	0.256^c^	0.228^c^	0.920^c^
NSS	1.6 ± 3.3	2.0 ± 3.1	3.8 ± 3.4	0.084^d^	> 0.999^b^	0.099^b^	0.636^b^
Sural NCV (m/s)	45.5 ± 4.6	47.1 ± 3.8	47.8 ± 7.5	0.397^d^	> 0.999^b^	0.539^b^	> 0.999^b^
Sural SNAP (μV)	8.5 ± 6.0	5.7 ± 3.7	5.2 ± 3.0	0.265^d^	0.657^b^	0.377^b^	> 0.999^b^
Peroneal nerve NCV (m/s)	45.3 ± 4.2	40.1 ± 6.3	42.3 ± 6.6	0.107^a^	0.117^c^	0.253^c^	0.341^c^
Peroneal nerve CMAP (mV)	9.0 ± 6.1	5.6 ± 2.4	5.9 ± 3.1	0.105^d^	0.295^b^	0.140^b^	> 0.999^b^
Peroneal nerve DML (ms)	5.5 ± 3.3	6.3 ± 4.5	5.8 ± 3.4	0.670^d^	> 0.999^b^	> 0.999^b^	> 0.999^b^
Tibial nerve NCV (m/s)	44.9 ± 4.0	41.2 ± 6.4	41.3 ± 6.7	0.170^a^	0.262^c^	0.218^c^	0.958^c^
Tibial nerve CMAP (mV)	15.8 ± 7.4	13.1 ± 8.2	12.6 ± 5.8	0.361^a^	0.563^c^	0.414^c^	0.846^c^
Tibial nerve DML (ms)	5.7 ± 4.2	6.0 ± 4.8	6.0 ± 4.5	0.636^d^	> 0.999^b^	> 0.999^b^	> 0.999^b^

^a^*p*-value obtained from ordinary one-way ANOVA, *BMI*, Body mass index; *CMAP*, Compound motor action potential

^b^*p*-value obtained from Kruskal–Wallis test with a post hoc Dunn procedure to correct for multiple comparisons, *DML* Distal motor latency, e*GFR* Estimated glomerular filtration rate

^c^*p*-value obtained from ordinary one-way ANOVA with Holm-Sidak’s procedure to correct for multiple comparisons

^d^*p*-value obtained from Kruskal–Wallis test

^e^*p*-value obtained from Mann–Whitney U test, *MTR* Magnetization transfer ratio, *NA* Not applicable, *NCV* Nerve conduction velocity, *NDS* Neuropathy disability score, *NSS* Neuropathy severity scale, *SNAP* Sensory nerve action potential

### Correlation analysis of MTR values with clinical and demographical parameters

In T1D patients, no correlations could be found for age, duration of diabetes, or BMI. The negative correlation of MTR with NDS score did not reach statistical significance (*r* = -0.56; *p* = 0.094). In T2D patients, MTR correlated negatively with age (*r* = -0.38; *p* = 0.047). A negative correlation with BMI (*r* = -0.36; *p* = 0.061) did not reach statistical significance. In HC, a weak correlation was found between the sciatic nerve’s MTR with BMI (*r* = -0.52; *p* = 0.059), which did not reach statistical significance in HC. For HC, no further correlations for sciatic nerve’s MTR with the compiled demographical and clinical data were found. A detailed summary of the results of correlation analysis is given in Table 3.

**Table 3:** Correlations of magnetization transfer ratio (MTR) with demographical, serological, clinical, and electrophysiological data

	MTR HC		MTR T1D		MTR T2D	
	*r*	*p*	*r*	*p*	*r*	*p*
Age	-0.21	0.482^b^	-0.22	0.548^a^	-0.38	0.047^a^
BMI (kg/m^2^)	-0.52	0.059^b^	-0.10	0.775^a^	-0.36	0.061^a^
Diabetes duration (years)	NA	NA	-0.12	0.734^a^	-0.29	0.147^b^
HbA1c (mmol/mol)	-0.01	0.964^b^	-0.63	< 0.050^a^	-0.17	0.383^a^
eGFR (mL/min)	0.24	0.141^b^	-0.18	0.713	-0.05	0.833^a^
NDS	-0.43	0.127^b^	-0.56	0.094^a^	0.26	0.211^a^
NSS	0.03	0.934^b^	-0.34	0.344^a^	0.04	0.853^b^
Sural NCV (m/s)	-0.10	0.711^b^	-0.02	0.988^b^	-0.50	0.034
Sural SNAP (μV)	0.30	0.276^b^	0.40	0.250^a^	-0.19	0.403
Peroneal nerve NCV (m/s)	0.05	0.857^b^	0.53	0.118^a^	-0.44	0.031^a^
Peroneal nerve CMAP (mV)	0.29	0.318^b^	0.16	0.661^a^	-0.11	0.612^a^
Peroneal nerve DML (ms)	-0.41	0.145^b^	0.13	0.713^b^	0.31	0.142^b^
Tibial nerve NCV (m/s)	0.33	0.250^b^	0.71	0.021^a^	-0.40	< 0.050^a^
Tibial nerve CMAP (mV)	0.17	0.553^b^	0.16	0.387^a^	-0.31	0.136^a^
Tibial nerve DML (ms)	0.07	0.805^b^	0.07	0.854^b^	0.13	0.539^b^

*BMI* Body mass index, *CMAP* Compound motor action potential, *DML* Distal motor latency, *GFR* Glomerular filtration rate, *MTR* Magnetization transfer ratio, *NA* Not applicable, *NCV* Nerve conduction velocity, *NDS* Neuropathy disability score, *NSS* Neuropathy severity scale

^a^*p*-value obtained from Pearson correlation analysis

^b^*p*-value value obtained from Spearman correlation analysis; *SNAP* Sensory nerve action potential

### Correlation analysis of MTR with serological and electrophysiological parameters

**Fig. 3:**
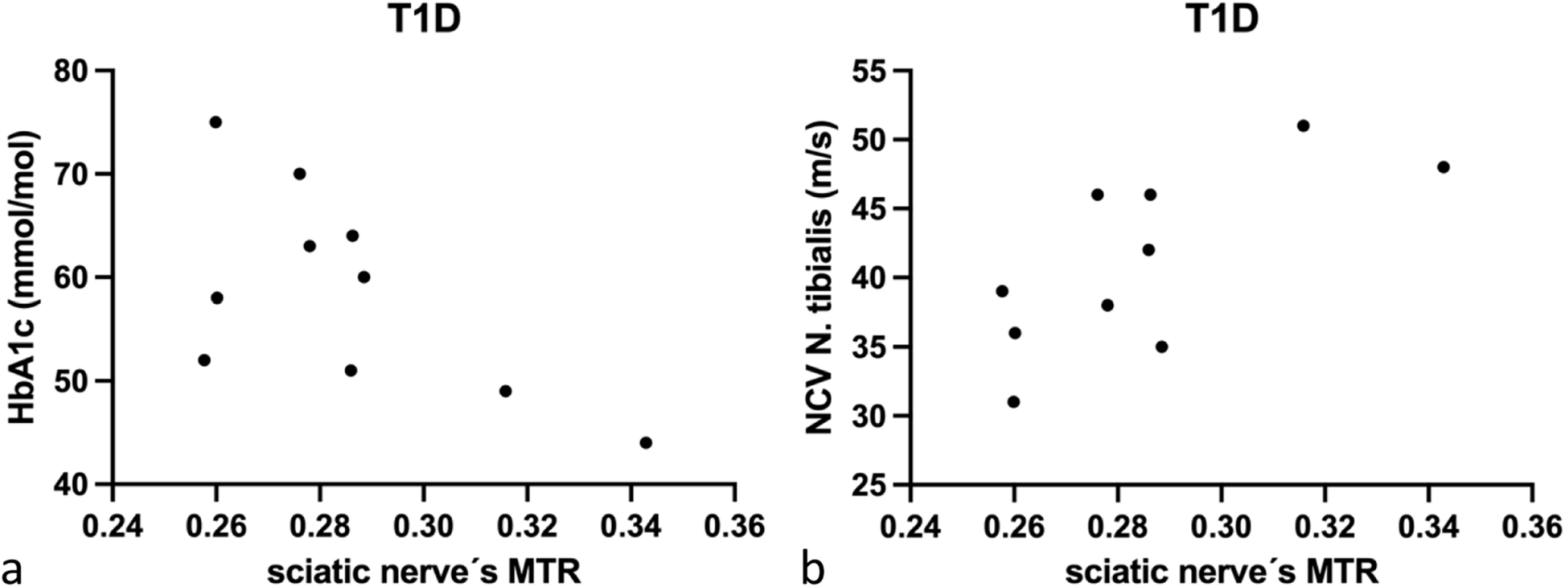
Correlation analysis of magnetization transfer ratio (MTR) of sciatic nerve with serological and electrophysiological parameters in patients with type 1 diabetes (T1D). **a** Correlation of sciatic nerve’s MTR with HbA1c (*r* = -0.63; *p* < 0.050). **b** Correlation of sciatic nerve’s MTR with tibial nerve conduction velocity (NCV; *r* = -0.71; *p* = 0.021)

**Fig. 4:**
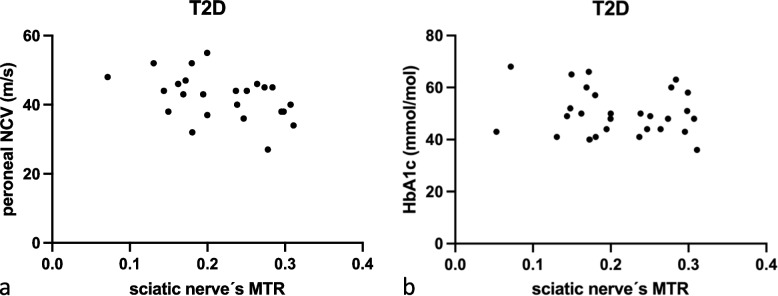
Correlation analysis of magnetization transfer ratio (MTR) of sciatic nerve with serological and electrophysiological parameters in patients with type 2 diabetes (T2D). **a** Correlation of peroneal nerve conduction velocity (NCV) with sciatic nerve’s MTR (*r* = -0.44; *p* = 0.031). **b** No correlations could be found between HbA1c with the sciatic nerve’s MTR (*r* = -0.17; *p* = 0.383)

In patients with T1D, HbA1c correlated negatively with MTR (*r* = -0.63; *p* < 0.050; Fig. 3a). Furthermore, tibial NCV correlated with MTR (*r* = 0.71; *p* = 0.021; Fig. 3b), while a correlation for peroneal NCV did not reach statistical significance (*r* = 0.53; *p* = 0.118). In patients with T2D, sural (*r* = -0.50; *p* = 0.034), tibial NCV (*r* = -0.40; *p* < 0.050), and peroneal (*r* = -0.44; *p* = 0.031; Fig. 4a) correlated negatively with sciatic MTR. Partial correlation analysis controlled for age and BMI between the sciatic nerve’s MTR and the peroneal NCV (*r* = 0.51; *p* = 0.016) remained significant, while sural NCV (*r* = 0.49; *p* = 0.054) and tibial NCV (*r* = 0.34; *p* = 0.115) failed to reach statistical significance. No correlations could be found for HbA1c (Fig. 4b). There were no significant correlations of sciatic nerve’s MTR with the compiled data in HC (*p* ≥ 0.059).

## Discussion

This study used 3-T MRN with MTR sequences to investigate potential differences of the sciatic nerve’s macromolecular composition between healthy controls and subjects with diabetes and to assess potential links of MTR parameters with clinical and electrophysiological parameters. The main findings were that (i) MTR of the sciatic nerve is lower in patients with T2D compared to patients with T1D and healthy controls; (ii) in T1D, MTR showed a positive correlation with tibial NCV, while, in T2D, MTR of the sciatic nerve correlated negatively with sural, peroneal, and tibial NCV; and (iii) in T1D, MTR correlated negatively with HbA1c, but not in T2D.

Our findings indicate that different pathophysiological mechanisms in T1D and T2D lead to distinct macromolecular changes in the peripheral nerves, which may be quantified with the use of MTR. In the context of different MRN lesion patterns in T1D and T2D DSN, with T2-weighted hyperintense nerve lesions linked to elevated glucose levels in T1D and T2-weighted hypointense lesions to lipid metabolism in T2D [7], this study supports the hypothesis that the underlying pathophysiological mechanisms of DSN differ between T1D and T2D [4, 5, 7]. In that regard, we only found HbA1c to be associated with the MTR of the sciatic nerve in patients with T1D, but not in T2D. We found HbA1c negatively and NCV of tibial nerve positively associated with MTR of sciatic nerve in patients with T1D, while NDS showed a trend of negative correlation with the sciatic nerve’s MTR (*p* = 0.094). These findings are in line with the fact that glycemic control is a key factor in preventing and treating DSN in T1D [4, 5] and that hyperglycemia is one of the main contributors to nerve damage in T1D [7].

Higher MTR values of the sciatic nerve were associated with structural nerve integrity in T1D patients. Meanwhile, in patients with T2D, we could not reproduce this finding, which agrees with the finding of previous studies that factors apart from hyperglycemia are the main contributors to nerve damage in patients with T2D [4, 5, 7]. However, the fact that fatty nerve lesions have been shown to predominate in T2D patients [7] may explain that MTR of the sciatic nerve is decreased in patients with T2D compared to patients with T1D and HC, as fatty tissues exhibit a low magnetization transfer [18, 32, 33]. It will be interesting to investigate which mechanisms are responsible for a similar sciatic MTR of HC and patients with T1D, especially because a previous study [26] was able to demonstrate that sciatic MTR was sensitive to discriminate between asymptomatic carriers of the mutant transthyretin gene causing hereditary transthyretin amyloidosis and HC.

The hypothesis, that nerve damage in T1D and T2D underlie different pathophysiological mechanisms, is further supported by the finding of opposed correlations of NCV with MTR of the sciatic nerve in T1D and T2D patients, respectively. The fact that MTR of the sciatic nerve showed correlations with NCV of the tibial and peroneal nerve indicates that MTR is a valuable marker of structural nerve integrity in T1D and T2D, as electrophysiological studies are considered to be the gold standard of assessing structural nerve integrity *in vivo* [34]. In T1D, MTR showed a positive correlation with tibial NCV, which agrees with the finding that lower MTR values were associated with a higher level of neurological deficits in patients with Charcot-Marie-Tooth disease, in hereditary transthyretin amyloidosis, and 5q spinal muscular atrophy [24–26]. As it is known that the lowering of NCV is generally an indicator of demyelinating nerve injury [35], one could hypothesize that a lowering of MTR in T1D might partly represent demyelinating nerve damage in T1D. Since this study found sciatic nerve MTR to be decreased in T2D compared to T1D and healthy controls, we hypothesize that the process of demyelination—one hallmark of DSN [36]—might be more pronounced in T2D. These assumptions are supported by previous studies on MTR of the central nervous system which proved a decrease of MTR to be a marker of demyelination [37, 38]. Conversely, the MTR of the sciatic nerve of amphibians [39] and the MTR of the optic nerve in patients with MS and optic neuritis [40] was associated not only with markers of demyelination but also of axonal nerve damage. These hypotheses remain to be demonstrated as we did not obtain specimens of peripheral nerves.

In contrast, our findings for T2D patients appear to be contradictory: while MTR is already lowered in T2D compared to HC, a further decrease would supposedly result in higher NCVs. One potential explanation for this may be that previous studies have shown that a low amount of structural nerve lesions in T2D patients does not cause changes that can be electrophysiologically detected, and that a higher amount of nerve lesions is required for patients to become symptomatic [41]. Thus, it is well possible that, in the T2D group, subclinical structural nerve damage is more present compared to the T1D group.

Since MTR has been shown to decrease with age and higher BMI in healthy subjects [23, 28], participants were matched for age and BMI to minimize the confounding impact of age. Also, no differences could be found between the T1D and T2D participants for NDS/NSS scores, HbA1c values, and electrophysiological parameters, rendering it unlikely that the observed differences between MTR in T1D and T2D are caused by a different severity of nerve damage or a difference in typical cofounding factors. The fact that T1D patients were diagnosed with disease a longer time ago than patients with T2D, and that MTR was only lower in T2D compared to T1D and HC, while T1D and HC did not differ, supports the hypothesis that different pathological pathways drive nerve damage in T1D and T2D. If nerve damage in T1D and T2D originated from the same mechanisms, we would expect MTR of T1D patients to be lower than that of T2D patients, since disease duration in T1D patients was significantly longer.

Our study is limited by several factors.

The cross-sectional design of this study does not allow a predictive assessment of the reported findings, which will be part of ongoing research. Moreover, future studies should correlate and compare T2-weighted hyperintense and hypointense nerve lesions with MTR and other MRN markers of DSN in T1D and T2D, to possibly gain more insights on the different nature of macromolecular changes and lesion types in both entities. It will especially be interesting to examine the outlined paradox of a lower MTR and its negative association with NCV in T2D.

The interpretation of the study results is also compromised by a lack of histological nerve samples. Subsequently, future research should focus on trying to correlate MRN findings with histological findings to explore these mechanisms and decode which histomorphological changes lead to alterations of proton spin density, T2 relaxation time, and T2-weighted hyperintense and hypointense nerve lesions as well as changes of MTR.

Another limitation represents the relatively small sample size of the cohort, which is why we cannot preclude all potential confounders through multivariate analysis. However, the patient groups did not show significant differences regarding important potential confounding factors such as age, BMI, and sex, and partial correlation analysis controlled for age and BMI, both potential confounders of MTR, was performed if needed. Also, the sample size did not allow conducting a comprehensive analysis for patients with and without DSN in T1D and T2D. Yet, patient groups did not differ regarding NSS, NDS, and parameters of nerve conductions studies, while it is also known that DSN represents a continuous process of accumulating nerve damage [41].

Another potential limitation is that only a relatively short segment of the sciatic nerve at the level of the distal thigh was examined. However, previous studies on MRN were able to demonstrate that nerve fiber damage predominates at the distal thigh and that MTR does not differ between proximal and distal thigh as well as lower leg [7, 23].

In summary, this study demonstrates that MTR imaging may provide a new imaging biomarker of structural damage/integrity of peripheral nerves in T1D and T2D. Our results underline that patterns of structural nerve damage and accompanied macromolecular changes differ in T1D and T2D patients. Consequently, these finding emphasize that different pathophysiological pathways drive nerve damage in T1D and T2D. Longitudinal studies applying MTR imaging are needed to decode these pathways in T1D and T2D.

## Data Availability

The datasets generated during and/or analyzed during the current study are not publicly available due to reasons of patient data protection but are available from the corresponding author upon reasonable request.

## Abbreviations

BMI: Body mass index
CMAP: Compound motor action potential
DML: Distal motor latency
DSN: Distal symmetric neuropathy
Hb1Ac: Hemoglobin A1c
HC: Healthy control
MRN: Magnetic resonance neurography
MTR: Magnetization transfer ratio
NCV: Nerve conduction velocity
NDS: Neuropathy disability score
NSS: Neuropathy severity scale
SNAP: Sural nerve action potential
T1D: Type 1 diabetes mellitus
T2D: Type 2 diabetes mellitus
